# A new analytical framework for multi-residue analysis of chemically diverse endocrine disruptors in complex environmental matrices utilising ultra-performance liquid chromatography coupled with high-resolution tandem quadrupole time-of-flight mass spectrometry

**DOI:** 10.1007/s00216-018-1483-y

**Published:** 2018-11-22

**Authors:** Luigi Lopardo, Axel Rydevik, Barbara Kasprzyk-Hordern

**Affiliations:** Department of Chemistry, University of Bath, Claverton Down, Bath, BA2 7AY UK

**Keywords:** Endocrine disruptor, Personal care products, Wastewater, Sludge, Environment, High-resolution LC-MS

## Abstract

This manuscript presents a comprehensive analytical framework for identification and quantification of chemically diverse endocrine disrupting chemicals (EDCs) used in personal care and consumer products in diverse solid and liquid environmental matrices with an ultimate goal of evaluating public exposure to EDCs via water fingerprinting. Liquid chromatography coupled with tandem quadrupole time-of-flight mass spectrometry (UHPLC-ESI-MS/MS) was used for targeted analysis of selected EDCs as well as to identify and quantify a few metabolites using post-acquisition data mining. Solid-phase extraction (SPE) was applied to liquid matrices in order to reduce matrix effects and provide required sample concentration and ultimately, high sensitivity and selectivity of measurements. SPE recoveries in liquid samples ranged from 49 to 140% with method quantification limits not exceeding 1 ng L^−1^ for the majority of EDCs. Microwave-assisted extraction (MAE) was applied to solid samples and when followed by SPE, it permitted the analysis of EDCs in digested sludge. MAE/SPE recoveries varied from 11 to 186% and MQLs between 0.03 and 8.1 ng g^−1^ with the majority of compounds showing MQLs below 2 ng g^−1^. Mass error for quantifier and qualifier ions was below 5 ppm when analysing river water and effluent wastewater and below 10 ppm when analysing influent wastewater and solid samples. The method was successfully applied to environmental samples, with 33 EDCs identified and quantified in wastewater and receiving waters. In addition, several EDCs were found in digested sludge, which confirms that for a more comprehensive understanding of exposure patterns and environmental impact, analysis of solids cannot be neglected. Finally, post-acquisition data mining permitted the identification and quantification of a metabolite of BPA and the identification of a metabolite of 4-Cl-3-methylphenol.

**Electronic supplementary material:**

The online version of this article (10.1007/s00216-018-1483-y) contains supplementary material, which is available to authorized users.

**Graphical abstract:**
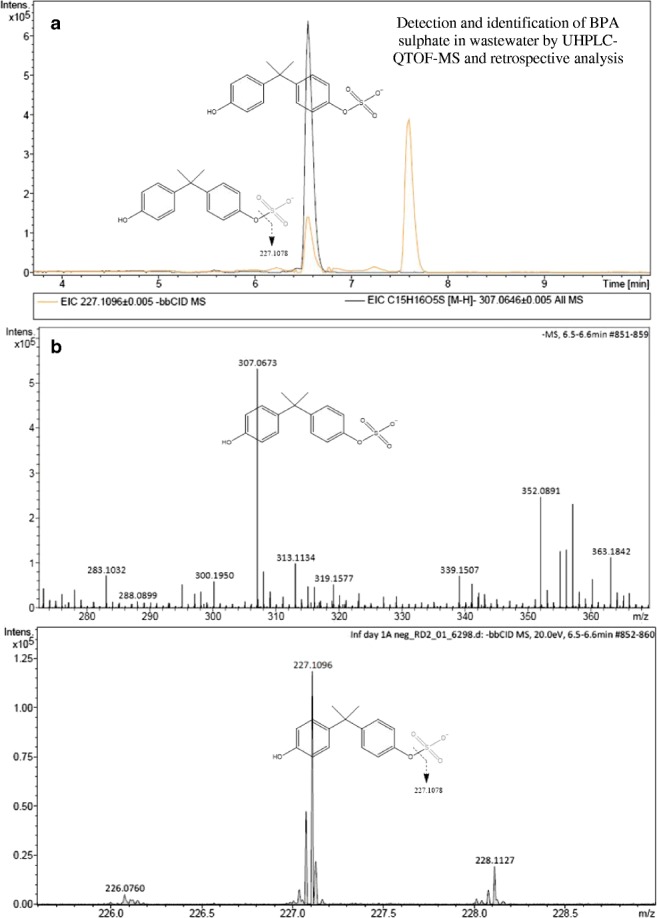
ᅟ

## Introduction

Many chemicals in personal care and consumer products such as preservatives, UV filters, plasticizers, fragrances, antimicrobials, pesticides and flame retardants are suspected to have or are recognised as endocrine system disruptors [1, 2]. Unfortunately, there is lack or limited understanding of the extent and patterns of human exposure to these chemicals. This is despite a critical need to obtain such data at a population level to inform future regulations.

Several papers have attributed serious health issues to different EDCs and calling for more research and regulations [3–6] but there are also some that argue that the problem is quite small when compared to natural hormone affecting compounds that are consumed with food, and that therefore think that such regulations would be unnecessary [7–9]. Therefore, accurate and reliable exposure assessments of EDCs are required if any more regulatory measures are to be taken, but it is difficult to do such an assessment at a population level.

Several studies have investigated the presence of EDCs in different types of samples including surface waters, wastewater, digested sludge and solid samples [10–19]. These studies have utilised different analytical techniques for both sample preparation, separation and detection. The most common method for sample preparation is solid-phase extraction (SPE), which has been used both online [12] and offline [10, 13, 14], but there are examples where stir bar sorptive extraction (SBSE) has been used instead [16]. A few of the studies have analysed their target analytes using gas chromatography (GC) coupled with mass spectrometer (MS) [10]. Others have used liquid chromatography (LC) coupled with MS [12–14]. Sosa-Ferrera et al. [20] compiled and compared different LC based methods to analyse various EDCs. However, most of these studies focussed on a small number of EDCs generally from a limited number of chemical classes [21–24] and/or are often investigated alongside other pharmaceuticals [14, 17, 19, 25–27]. Moreover, these studies used targeted MS detection meaning that their results cannot be used for retrospective data analysis.

The aim of this work was to develop a robust analytical framework for selective and sensitive multi-residue analysis of structurally diverse EDCs (ranging from fragrances to brominated flame retardants) in wastewater (both solid and liquid samples) and receiving environment which includes the capability to undertake retrospective data mining on analysed samples. To achieve this, ultra-high performance liquid chromatography (UHPLC) coupled with high-resolution mass spectrometry (HRMS) was used for targeted analysis of EDCs and post-acquisition identification and quantification of their known metabolites as well as identification of newly discovered metabolites.

## Materials and methods

### Chemicals

The following analytes were targeted in this study (see Electronic Supplementary Material (ESM) Table S1): 2,4,5-trichlorophenol, 2,4,6-trichlorophenol, 2-ethylhexanoic acid, 2-naphthol, 4,4′-dihydroxybenzophenone, 4-benzylphenol, 4-chloro-3,5,dimethylphenol, 4-chloro-3-methylphenol, 4-n-nonylphenol, 4-n-octylphenol, atrazine, benzophenone-1 (BP-1), benzophenone-2 (BP-2), benzophenone-3 (BP-3), benzophenone-4 (BP-4), benzylparaben, bisphenol A (BPA), bisphenol A bis(3-chloro-2-hydroxypropyl) ether (BADGE-2-Cl), butylparaben, chlorothymol, dibutyl phthalate (DBP), ethylparaben, galaxolide, 1,2,5,6,9,10-hexabromo-cyclododecane (HBCD), mono(2-ethylhexyl)phthalate (MEHP), methylparaben, monobutyl phthalate (MBP), musk ketone, padimate O, perfluorooctanesulfonic acid (PFOS), perfluorooctanoic acid (PFOA), phenylbenzimidazolesulfonic acid (PBSA, ensulizole), prochloraz, propylparaben, tetrabromobisphenol A (TBBPA), triclocarban, triclosan and vinclozolin (see ESM Table S1 for further details). The internal standards used were: 4-chloro-3-methylphenol-d_2_, atrazine-d_5_, bezafibrate-d_6_, BP-3-d_5,_ triclosan d_3_ and triclocarban-d_4_ (QMX (UK) or TRC (UK). Water was purified using a Milli-Q purification system from Millipore (Nottingham, UK). Methanol, formic acid (> 95%), HCl (concentrated), 1 M NaOH, 1M NH_4_OH, NH_4_F and 2-propanol were purchased from Sigma (UK) and Fisher (UK). All solvents used were of LC grade or higher.

Glassware was deactivated using 5% dimethyldichlorosilane in toluene (DMDCS; Sigma, UK) to prevent losses from analyte adsorption. The deactivation procedure consisted of washing the glassware once with 5% DMDCS followed by two washes with toluene and lastly three washes with methanol.

### Sample collection

Pooled influent and effluent wastewater samples were collected at a wastewater treatment plant using ISCO 3700 portable samplers (RS Hydro, Worcestershire, UK) that were set up to do volume proportional collections of 10 mL portions with an average sampling rate of 15 min. The samples were kept at 4 °C until collection and transported on ice to the laboratory. After spiking with internal standards, pooled 24-h samples were then stored at − 18 °C until sample preparation and analysis. Grab samples were also collected from receiving river waters from upstream and downstream of the effluent discharge point on each sampling day. Digested sludge was collected from an anaerobic digestion plant.

### Sample preparation

#### Liquid matrix—solid-phase extraction

One hundred millilitres of each sample was transferred to 125-mL plastic bottles (HDPE) and spiked with 25 μL of an internal standard mixture (100 μg L^−1^). After spiking, the samples (100 mL) were filtered using GF/F (0.7 μm) filters (Whatman, UK) and extracted using SPE. After initial screening using Oasis HLB, MCX and MAX sorbents, HLB sorbent was selected for further study. HLB extraction protocol included conditioning of 60 mg 3 cc HLB cartridges (Waters, UK) with 2 mL of methanol followed by 2 mL of water. Samples were adjusted to a neutral pH with formic acid (> 95%) or 1 M NaOH and then applied to HLB cartridges using vacuum at a flow rate of 3 mL min^−1^. After a 30-min drying step, all cartridges were stored in a freezer at − 18 °C until elution. Elution was undertaken using 4 mL of methanol. All eluates were evaporated under a stream of nitrogen at 40 °C in a water bath (TurboVap evaporator (Calliper, UK)) and then reconstituted with 250 μL of H_2_O:MeOH 80:20. Samples (90 μL) were then injected on the UHPLC-QTOF.

#### Solid matrix—microwave-assisted extraction

Microwave-assisted extraction was performed according to the procedure described in Petrie et al., 2015. After collection, digested sludge samples were frozen and then freeze dried using a ScanVac, CoolSafe freeze dryer (Lynge, Denmark). One hour before the extraction, 50 ng of each internal standard was added to 0.5 g of digested sludge and extraction was performed using 25 mL of 50:50 MeOH:H_2_O (pH 2) using a 800 W MARS 6 microwave (CEM, UK). Samples were heated at 110 °C for 30 min. After extraction, samples were filtered using GF/F filters (0.7 μm) and the content of MeOH was diluted to < 5% using H_2_O (pH 2). Finally, samples were loaded at 5 mL min^−1^ onto Oasis MCX cartridges conditioned with 2 mL of MeOH followed by 2 mL of H_2_O (pH 2) at 1 mL min^−1^. MCX cartridges were then dried for 30 min and analytes were then eluted in separate acidic and basic fractions. For further detail, see Petrie et al. [15].

### Analysis

#### Targeted UHPLC-QTOF analysis for selected EDCs: full structural confirmation and quantification with commercially available reference standards

The analysis was performed on a Dionex UltiMate3000 UHPLC system (Thermo Fisher UK Ltd.) connected to a maXis HD Q-ToF mass spectrometer (Bruker, UK) with mass resolution of 45,000 and controlled by the Compass software (HyStar™ Bruker, UK). Ninety microlitres of analyte standard solutions (see section Chemicals) and SPE extracted environmental samples were injected onto a BEH C18 column (50 × 2.1 mm, 1.7 μm, Waters UK) at a flow rate of 0.4 mL min^−1^.

##### Mobile phase composition

Initial experiments revealed that mobile phase composed of 0.1% formic acid (FA) in water and methanol (linear gradient) did not provide satisfactory separation of all analytes and did not facilitate satisfactory signal intensities in negative ionisation mode (e.g. BPA). On the other hand, 1 mM NH_4_F in water as the aqueous phase resulted in better signal intensities in negative ionisation mode but it also resulted in lower signal intensities in positive ionisation mode. As the aim was to have one separation method serving both positive and negative ionisation modes, the gain in signal intensities in negative mode was weighed against the loss in signal intensities in positive mode. Since more analytes were detected using mobile phase containing 1 mM NH_4_F and the gain in signal intensities in negative mode was higher than the number of analytes and the loss of signal in positive mode, this mobile phase was selected for further experiments.

The best separation of all analytes was achieved using the following gradient: mobile phase A (1 mM NH_4_F in water) and mobile phase B (methanol) at the following gradient: 0–3 min 5 %B, 3–4 min 5–60 %B, 4–14 min 60 %B, 14–14.1 min 60–98 %B, 14.1–17 min 98 %B, 17–17.1 min 98–5 %B, 17.1–20 min 5 %B).

##### Mass spectrometry parameters

Instrument mass calibration was performed by an injection of 10 μL of a calibrant solution (3 parts of 1 M NaOH to 97 parts of 50:50 water:IPA with 2% FA) at the start of each run before the sample injection. The resulting peak was used for internal mass calibration using the software DataAnalysis (Compass DataAnalysis 4.3 Bruker, UK).

The mass spectrometer was equipped with an ESI source and was operated in both positive and negative ionisation mode. A capillary voltage was set at 4.5 kV, the end plate offset was set to 500 V, a pressure of 3 Bar was used for the nebuliser gas, the drying gas (nitrogen) flow was 11 L min^−1^ and the drying temperature was set at 220 °C. The bb(broad band)CID settings in negative mode were 0 eV of isCID energy in both MS and MS/MS while the respective collision energies were 7 and 20 eV. The bbCID settings in positive mode were 0 eV of isCID energy in both MS and MS/MS while the respective collision energies were 5 and 20 eV.

Collected data was processed using DataAnalysis and QuantAnalysis (Bruker, UK). The method was then fully validated using the following:

##### Extraction recovery

Three complementary SPE chemistries (HLB, MCX and MAX) were used to cover as large a spectrum of analytes as possible since the method was to be also used for retrospective analysis of analytes not yet targeted. Oasis HLB sorbents showed the highest SPE recoveries in all studied matrices and therefore HLB sorbent was selected for further study. Due to co-elution, 2,4,5-trichlorophenol and 2,4,6-trichlorophenol were evaluated together as one peak.

The method extraction recovery was evaluated by spiking 100 mL of influent and effluent wastewater and river water in triplicate at two different analyte concentrations: 100 and 200 ng L^−1^ and internal standards: 100 ng L^−1^ after SPE.

Method recoveries were calculated as corrected recoveries (i.e. taking the internal standard concentration into consideration) by the ratio of the concentration of target analytes in wastewater solutions when spiked before SPE (minus the concentration of analyte in the blank wastewater sample), divided by the standard mobile phase concentration (Eq. 1).

1 $$ {\mathrm{Method}\ \mathrm{Recoveries}}_{\mathrm{corrected}}=\left(\frac{{\mathrm{A}}_{\mathrm{spiked}\ \mathrm{before}\ \mathrm{SPE}}-{\mathrm{A}}_{\mathrm{blank}}}{{\mathrm{A}}_{\mathrm{mobile}\ \mathrm{phase}}}\right)\times 100\% $$

##### Choice of internal standards

Internal standards for each analyte were chosen based on 4 different criteria. If an isotopically labelled analogue was available, then that was the internal standard of choice. If a labelled standard was not available, then the internal standard was chosen by evaluating several labelled compounds in terms of extraction efficiency, retention times and analyte vs internal standard area ratios stability in pure water and wastewater. The internal standards chosen are presented in Table 1.

**Table 1:** UHPLC-QTOF instrument performance parameters

Analyte	Internal standard used	ESI	Rt [min]	Linearity		Intra-day instrument performance^1, 2^		Inter-day instrument performance		IDL [μg L^−1^]	IQL [μg L^−1^]^3^
				Range [μg L^−1^]	*R* ^2^	Accuracy [%]	Precision [%]	Accuracy [%]	Precision [%]		
2,4,5 & 2,4,6-trichlorophenol	Triclosan-d3	neg	9.1	0.23–69.5	0.9989	92.6	8.5	90.1	12.7	0.1	0.3
2-ethylhexanoic acid*^4^	Triclosan-d3	neg	7.9	4–36.5	0.9978	/	/	/	/	/	/
2-naphthol	Bezafibrate-d6	neg	7.2	0.09–36	0.9985	95.7	10.8	77.8	17.9	0.03	0.09
4,4′-dihydroxybenzophenone	Bezafibrate-d6	neg	6.6	0.008–21.5	0.9994	109.2	11.4	95.2	7.8	0.02	0.08
4-benzylphenol	Bezafibrate-d6	neg	8.3	0.1–55	0.9995	138.8	9.8	136.6	19.4	0.03	0.1
4-chloro-3,5-dimethylphenol	4-Cl-3-methylphenol-d2	neg	8.2	0.04–27.5	0.9995	103.4	11.8	109.7	5.1	0.01	0.04
4-chloro-3-methylphenol	4-Cl-3-methylphenol-d2	neg	7.5	0.06–27.5	0.9987	120.2	2.4	120.2	3.5	0.02	0.06
4-n-nonylphenol	Triclosan-d3	neg	17.3	0.6–63	0.9984	123.71	4.8	124.1	8.1	0.2	0.6
4-n-octylphenol*	4-Cl-3-methylphenol-d2	neg	17	0.1–56	0.9954	/	/	/	/	/	/
Atrazine	Atrazine-d5	pos	7.5	9.9–99	0.9984	102.1	1.6	102.1	3.3	0.01	0.03
Benzophenone-1	4-Cl-3-methylphenol-d2	neg	7.8	0.1–31.8	0.9975	110.4	8.3	110.4	15.0	0.03	0.1
Benzophenone-2	Bezafibrate-d6	neg	6.8	0.1–88	0.9996	98.0	6.5	98.0	10.1	0.04	0.1
Benzophenone-3	BP-3-d5	pos	9.2	0.6–54	0.9992	104.4	8.4	113.5	4.2	0.2	0.6
Benzophenone-4	Bezafibrate-d6	neg	6.5	0.1–11	0.9972	98.5	5.1	98.5	19.5	0.04	0.1
Benzylparaben	Bezafibrate-d6	neg	8.3	0.07–33	0.9998	74.8	8.9	74.8	19.3	0.02	0.07
Bisphenol A	4-Cl-3-methylphenol-d2	neg	7.7	0.3–28.5	0.9972	114.8	2.5	114.8	9.4	0.08	0.3
BADGE-2-Cl	4-Cl-3-methylphenol-d2	neg	13.1	0.1–4.7	0.9989	103.4	7.1	103.4	19.9	0.03	0.1
Butylparaben	Bezafibrate-d6	neg	8.3	0.1–32	0.9994	93.4	8.8	93.4	15.6	0.04	0.1
Chlorothymol	Triclosan-d3	neg	10.7	0.1–48.5	0.9974	118.0	2.8	135.8	12.3	0.03	0.1
dibutyl phthalate	Atrazine-d5	pos	13.8	1.0–96	0.9899	81.3	3.0	80.5	3.3	0.3	1.0
Ethyl paraben	Bezafibrate-d6	neg	7.0	0.3–100.5	0.9993	92.9	5.7	92.9	9.0	0.08	0.3
Galaxolide	Atrazine-d5	pos	17.1	0.5–51.5	0.9967	94.6	16.3	91.8	18.0	0.1	0.5
HBCD*	Bezafibrate-d6	neg	17.5	0.1–10.5	0.9966	/	/	/	/	/	/
MEHP	4-Cl-3-methylphenol-d2	neg	8.7	0.3–5.1	0.9952	90.4	7.4	90.4	19.6	0.08	0.3
Methylparaben	Bezafibrate-d6	neg	6.6	0.4–30.5	0.9990	131.7	10.8	131.7	16.3	0.1	0.4
Monobutyl phthalate	4-Cl-3-methylphenol-d2	neg	6.7	0.2–39	0.9969	130.3	16.4	130.3	19.0	0.05	0.2
Musk ketone	4-Cl-3-methylphenol-d2	neg	12	0.5–27.5	0.9952	129.9	9.1	94	12.2	0.2	0.5
Padimate O	Atrazine-d5	pos	17.3	0.4–27.4	0.9974	93.6	14.7	86.8	16.9	0.1	0.4
Perfluorooctanesulfonic acid	Triclocarban-d4	neg	8.9	1.0–53	0.9993	98.9	7.2	99.2	18.6	0.3	1.0
Perfluorooctanoic acid	Triclocarban-d4	neg	8.2	1.4–104.5	0.9993	117.3	2.7	117.3	4.1	0.4	1.4
PBSA	Triclosan-d3	neg	6.1	0.2–11	0.9974	94.0	5.8	94.0	20.0	0.05	0.2
Prochloraz*	BP-3-d5	pos	11.6	0.3–12.7	0.9970	122.1	14.4	133.9	15.4	0.09	0.3
Propylparaben	4-Cl-3-methylphenol-d2	neg	7.5	0.4–31.5	0.9996	122.0	6.3	122.0	15.7	0.1	0.4
Tetrabromobisphenol A	Triclocarban-d4	neg	15.6	0.1–12	0.9970	171.2	9.3	171.2	9.4	0.04	0.1
Triclocarban	Triclocarban-d4	neg	13.5	0.1–27.5	0.9993	114.5	3.5	114.5	4.6	0.03	0.1
Triclosan	Triclosan-d3	neg	14.6	0.1–53.2	0.9997	102.1	2.9	102.1	7.0	0.03	0.1
Vinclozolin	4-Cl-3-methylphenol-d2	neg	11.2	1.1–88	0.9972	104.8	14.4	104.8	19.4	0.3	1.1
Solid analysis											
2,4,5 and 2,4,6-trichlorophenol	4-Cl-3-methylphenol-d2	neg	9.1	0.3–69.5	0.9980	83.2	8.1	83.2	19.0	0.08	0.3
4-n-nonylphenol	4-Cl-3-methylphenol-d2	neg	17.3	0.6–63	0.9972	123.71	4.8	128.3	11.6	0.2	0.6
Chlorothymol	4-Cl-3-methylphenol-d2	neg	10.7	0.3–48.5	0.9971	118.7	3.0	138.3	14.5	0.1	0.3
PBSA	4-Cl-3-methylphenol-d2	neg	6.1	0.27–11	0.9969	111.9	6.0	94.1	19.5	0.1	0.3
Triclosan	4-Cl-3-methylphenol-d2	neg	14.6	0.29–53	0.9997	122.8	2.8	122.8	7.8	0.1	0.3

Note: 1: concentration levels: 0.1, 5 and 100 ng/mL used for inter- intraday precision and accuracy (only the levels within the linear range were used for each compound). 2: 5 replicates were injected of each concentration level for each day. 3: IQL is set as the lowest linear point that has a precision of < ± 20%. 4; starred compounds denoted quality control criteria out of the acceptance range suggested by the European commission

##### Linearity

The linearity in detector’s response for each analyte was evaluated by the construction of calibration curves from 13 different concentration levels (ranging from 0.01 to 100 μg L^−1^) in mobile phase. The linearity was interpreted as the *R*^2^ for the resulting linear regressions (based on all concentration levels or a selection thereof depending on the analyte).

##### Instrument and method limits of detection and quantification

The instrument detection and quantification limits (IDL and IQL respectively) were evaluated through the use of the calibration curves to calculate the concentrations that gave signal-to-noise ratios of 3 and 10 respectively. The IDLs and IQLs were then used to calculate the method detection and quantification limits (MDL and MQL respectively) by using Eq. 2.

2 $$ ML=\frac{IL}{400}\ast \frac{1}{RC} $$

ML and IL stand for MDL/MQL and IDL/IQL respectively. Four hundred is the concentration factor due to SPE and RC is the SPE recovery. When the calculated MQL was lower than the lowest concentration used in the calibration curve, then the MQL was set to match the calibration curve.

##### Inter- and intraday precision

Inter- and intraday precision for the instrument was evaluated from QC-standards up to three concentrations (0.1, 5 and 100 μg L^−1^) injected in triplicate. Standards that were within the linear range of the analyte were used for the calculations. The inter- and intraday precision for the method was evaluated by spiking wastewater and river water at two different concentrations (100 and 200 ng L^−1^) followed by extraction in triplicate on three consecutive days (total *n* = 9) using HLB cartridges.

##### Accuracy

The accuracy was assessed by comparing calculated concentrations using established calibration curves with the theoretical concentrations and calculating the average error with its associated RSD.

#### Post-acquisition data mining for metabolite identification and quantification

The collection of full-scan spectra permits to measure compounds not included in a target list leading to the possibility of retrospective data analysis, and the capability of performing structural elucidations and quantification of unknown or suspect compounds. The level system approach utilised in this paper to identify and quantify metabolites with various levels of confidence was modified from Schymanski et al. [28]. Two confidence levels were investigated: level 1a (structure confirmed by commercially available reference standards followed by full quantification and level 1b (structure confirmed by reference compounds synthesised in vitro*,* (see Lopardo et al. [29] for details)) with minimum identification criteria required being: i) lower retention time then their respective parent compound given their lower lipophilicity; ii) high mass accuracy (mass error below 10 ppm); iii) isotope pattern matching the predicted one (within 5% error).

In level 1a, the proposed structure of metabolites was confirmed via the utilisation of commercial reference standards in both MS and MS/MS mode, as well as matching retention time. This approach allowed for full quantification.

In level 1b, an exact structure of metabolites was proposed using in vitro HLM/S9 fraction assays (see Lopardo et al. [29] for details) as evidence. The proposed structure of metabolites was confirmed by comparing both MS and MS/MS mode and matching retention time to the in vitro produced metabolite as well as in vivo products in pooled urine and wastewater.

## Results and discussion

**Fig. 1:**
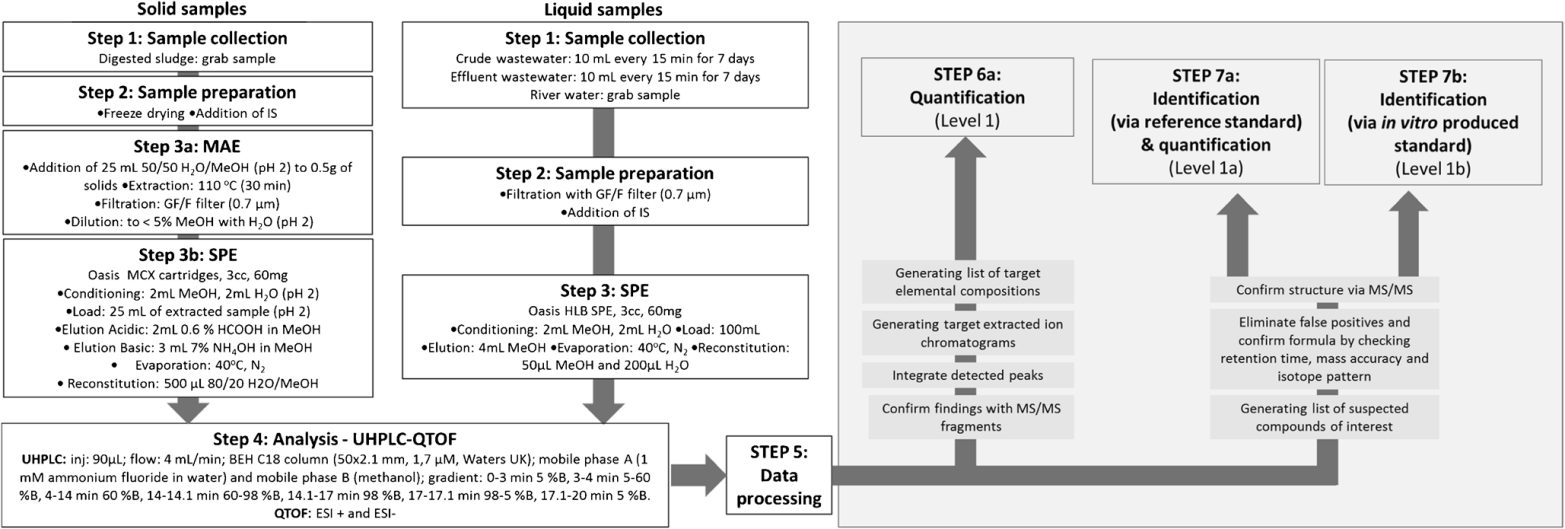
Schematic overview of the analytical protocol used to investigate the presence of EDCs in environmental matrices

The aim of this study was to develop a sensitive and selective multi-residue method for both quantification of trace concentrations of EDCs in wastewater and in the receiving environment and a posteriori analysis of metabolites of interest by data mining. Figure 1 shows the workflow of the followed analytical procedure, including determination of targeted compounds and data mining.

### Targeted UHPLC-QTOF analysis for selected EDCs: full structural confirmation and quantification with commercially available reference standards

#### MAE/SPE-UHPLC-QTOF method validation

##### Mass spectrometry parameters

**Fig. 2:**
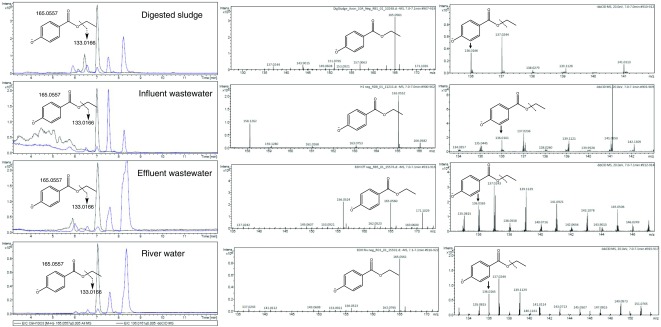
Separation and identification of ethylparaben in all analysed matrices (influent and effluent wastewater, river water and digested sludge). XIC at m/z 165.0557 (0.005-Da mass-window width, black trace) and at m/z 136.0166 (0.005-Da mass-window width, blue trace) in four different matrices (from top to bottom), and respective low-energy and high-energy mass spectra (from left to right) of the peak eluted at 7.0 min

**Fig. 3:**
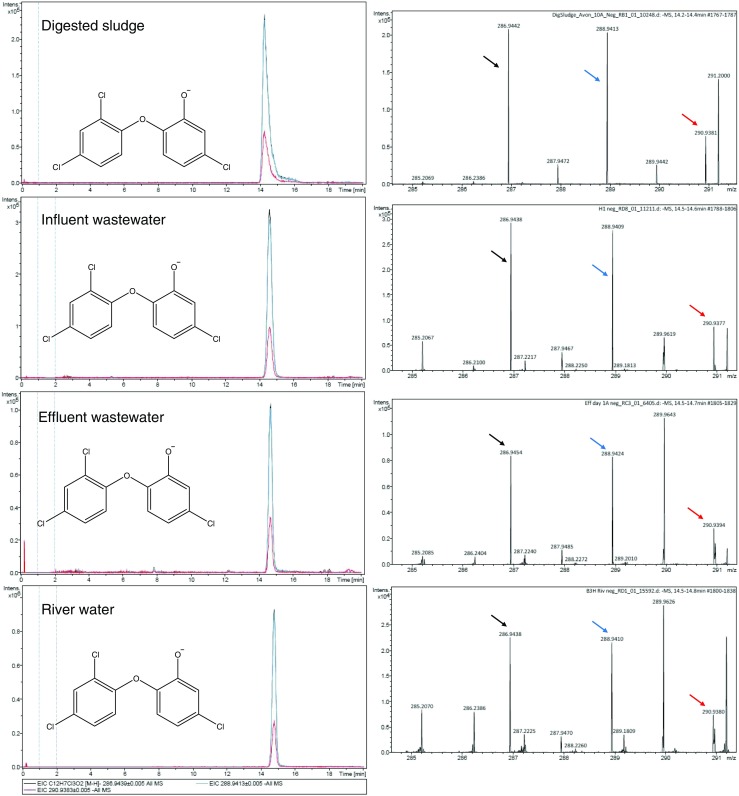
Separation and identification of triclosan in all analysed matrices (influent and effluent wastewater, river water and digested sludge). XIC at m/z 286.9439 (0.005-Da mass-window width, black trace), at m/z 288.9413 (0.005-Da mass-window width, blue trace) and at m/z 290.9383 (0.005-Da mass-window width, red trace) in four different matrices (from top to bottom). The right column shows the mass spectra of the peak eluted at 14.6 min and the black, blue and red arrows indicate respectively [M]^−^, [M + 2] ^-^, [M + 4] ^–^ peaks with relative intensities matching those expected from a compound with three chlorines within 5% of the predicted abundance

MS/MS parameters were optimised for all analytes and their corresponding labelled internal standards (for details see Table 1). Of the 37 compounds investigated, only 6 showed better sensitivity in ESI positive mode, while the vast majority provided better sensitivity in ESI negative mode. One product ion was monitored for a given collision energy for each precursor ([M + H]^+^ or [M-H]^−^). High mass accuracy (< 10 ppm mass error) for quantifier, qualifiers and isotope ions was used as criteria for identification and quantification purposes according to the EU guidelines [30]. For all compounds, the molecular mass plus/minus a hydrogen ion was selected as quantifier ion, while one product ion at higher collision energy was monitored in complex matrices for confirmation purposes. Unfortunately, due to limited fragmentation, no products could be monitored for 3 compounds (BADGE-2-Cl, HBCD and triclosan). Nonetheless, the level of certainty for those compounds is still high due to the presence of multiple halogens in their chemical structures, meaning complex and highly distinctive isotope patterns. Retention time and ion ratio within the standard tolerance were also monitored to ensure the quality of the data. Examples of two compounds’ (methylparaben and triclosan) identification in environmental matrices are reported in Figs. 2 and 3.

##### Solid-phase extraction

Three types of SPE cartridges were tested: Oasis HLB, MCX and MAX. HLB gave good relative recoveries for most compounds with the 54% of analytes having recoveries between 80 and 110% (Table 2). The difference in recoveries between high and low spiking levels was mostly below 10% RSD. HLB recoveries were also evaluated at two different levels of spiking in effluent wastewater and river water. Relative recoveries for effluent wastewater were in between 80 and 110% for 68% of the compounds with relative recovery difference between high and low spiking level mostly below 10% RSD. Relative recoveries were better when SPE was performed on river water samples due to the lower complexity of the matrix. Eighty-four percent of the values were between 80 and 110% with relative recovery difference between high and low spiking level mostly below 10% RSD.

**Table 2:** SPE-UHPLC-QTOF method performance parameters

Analyte	Wastewater influent			Wastewater effluent			River water			Digested sludge		
	SPE recovery [%]^1^	MDL [ng L^−1^]	MQL [ng L^−1^]	SPE recovery [%]	MDL [ng L^−1^]	MQL [ng L^−1^]	SPE recovery [%]	MDL [ng L^−1^]	MQL [ng L^−1^]	MAE recovery [%]	MDL [ng g^−1^]	MQL [ng g^−1^]
2,4,5 and 2,4,6-trichlorophenol	94.4	0.2	0.7	89.5	0.2	0.9	101.9	0.2	0.7	66.5	0.31	1.1
2-naphthol*^3^	140.7	0.06	0.2	91.4	0.07	0.3	109.8	0.1	0.2	/	/	/
4,4′-dihydroxybenzophenone	106.6	0.06	0.2	100.6	0.06	0.2	109.5	0.1	0.2	59.7	0.01	0.03
4-benzylphenol*	105.2	0.1	0.3	80.1	0.1	0.3	100.9	0.1	0.3	/	/	/
4-chloro-3,5-dimethylphenol	90.8	0.04	0.1	80.7	0.04	0.12	101.5	0.03	0.1	131.9	0.02	0.08
4-chloro-3-methylphenol	81.5	0.1	0.3	89.9	0.05	0.18	105.6	0.05	0.15	68.9	0.07	0.2
4-n-nonylphenol	49.1	0.9	2.9	43.8	1.0	3.4	67.7	0.7	2.2	18.4	2.5	8.2
Atrazine	99.0	0.03	0.09	94.2	0.03	0.09	102.6	0.02	0.08	68.4	0.01	0.04
Benzophenone-1	89.7	0.1	0.3	107.9	0.08	0.3	102.0	0.08	0.3	69.2	0.1	0.4
Benzophenone-2	102.9	0.1	0.3	98.9	0.09	0.3	106.6	0.08	0.3	75.5	0.1	0.4
Benzophenone-3	86.1	0.5	1.7	80.4	0.6	1.8	87.2	0.5	1.7	186.2	0.2	0.8
Benzophenone-4	105.3	0.1	0.3	80.1	0.1	0.4	105.1	0.1	0.3	17.7	0.6	2.0
Benzylparaben	122.4	0.04	0.1	100.1	0.05	0.2	99.7	0.05	0.2	145.9	0.03	0.1
Bisphenol A	100.4	0.2	0.7	64.2	0.3	1.0	86.0	0.2	0.8	95.7	0.2	0.7
BADGE-2-Cl	122.9	0.06	0.2	73.5	0.1	0.3	81.8	0.1	0.3	24.6	0.3	1.0
Butylparaben*	105.3	0.1	0.3	95.1	0.1	0.3	107.4	0.1	0.3	/	/	/
Chlorothymol	84.5	0.2	0.7	88.4	0.2	0.7	96.1	0.2	0.7	101.4	0.2	0.6
Dibutyl phthalate*	71.2	1.0	3.4	93.6	0.8	2.6	95.1	0.8	2.5	/	/	/
Ethylparaben	87.9	0.2	0.8	108.4	0.2	0.6	107.1	0.2	0.6	112.1	0.2	0.6
Galaxolide*	50.7	0.7	2.2	100.8	0.3	1.1	102.1	0.3	1.1	/	/	/
MEHP*	110.5	0.2	0.6	82.4	0.2	0.8	95.6	0.2	0.7	61.6	0.3	1.1
Methylparaben	80.5	0.3	1.1	109.3	0.2	0.8	104.6	0.3	0.8	86.4	0.3	1.0
Monobutyl phthalate	84.2	0.1	0.5	84.9	0.1	0.5	87.1	0.1	0.5	26.3	0.5	1.5
Musk ketone*	73.9	0.5	1.8	72.1	0.6	1.8	81.7	0.5	1.6	/	/	/
Padimate O*	49.8	0.6	2.0	28.8	1.0	3.4	53.7	0.6	1.8	/	/	/
Perfluorooctanesulfonic acid*	89.6	0.7	2.4	84.7	0.9	2.8	69.1	1.0	3.5	/	/	/
Perfluorooctanoic acid	101.7	1.2	3.8	92.3	1.1	3.7	95.4	1.1	3.6	18.4	5.6	18.6
PBSA	138.5	0.1	0.3	73.5	0.2	0.5	71.4	0.1	0.4	20.1	0.6	2.0
Prochloraz*	95.7	0.2	0.8	83.8	0.3	1.0	107.0	0.2	0.7	/	/	/
Propylparaben	89.2	0.3	1.1	112.9	0.3	0.8	93.5	0.3	1.0	86.4	0.3	1.1
Tetrabromobisphenol A	88.3	0.1	0.4	71.0	0.2	0.5	83.8	0.1	0.4	11.3	1.0	3.2
Triclocarban*	70.7	0.1	0.4	61.9	0.1	0.4	73.4	0.1	0.4	/	/	/
Triclosan*	73.8	0.1	0.4	80.4	0.1	0.3	82.8	0.1	0.3	/	/	/
Vinclozolin*	33.0	2.5	8.3	5.0	16.5	55.0	4.99	16.5	55.1	/	/	/

Note: 1: based on triplicate extractions. 2: based on triplicate injections at three concentration levels (*n* = 9), for some analytes one injection had to be removed making *n* = 8 3: starred compounds showed poor or no recovery from solid samples

##### MAE/SPE recoveries

A microwave-assisted extraction (MAE) method developed by Petrie et al. [15] was selected to prepare solid samples as it provided good recoveries for over 60 of the 90 compounds investigated in the study, including some endocrine disruptors selected in the present work such as BP-1, BP-2, BP-4, BPA, triclosan and methyl-, ethyl-, propyl- and butylparaben. In this study, MAE/SPE recoveries ranged from 11 to 186% with the majority of compounds denoting recoveries between 59 and 115% (Table 2). However, 8 compounds could not be analysed with the current method due to poor SPE recoveries, poor MAE efficiency or a combination of the two. More specific targeted analytical methods are therefore necessary for these EDCs.

##### Inter- and intraday accuracy and precision

Intra- and inter-day accuracy was typically within the range 80–130% for most chemicals both within the same day and between different days (Table 1). Instrumental intra- and inter-day precision calculated for three concentration levels at three consecutive days denoted on average 8% and 13% (Table 1). The precision of the method was also evaluated at three different concentration levels that were extracted in triplicate using HLB on three consecutive days (giving a total *n* = 9). The results from the evaluation are presented in Table 2. Overall, the intraday precision of the method is good with the majority of analytes giving precision values below 10% RSD in both positive and negative mode with the highest value being 18.4% RSD. For inter-day precision, the spread was a little bit higher with all analytes giving less than 20% RSD. Three compounds (2-ethylhexanoic acid, 4-n-octylphenol, HBCD) resulted in satisfactory linearity across studied concentration ranges but showed poor accuracy and precision. The high variability was due to poor ionisation rate in the selected analytical conditions. Their analysis can therefore be considered only qualitative.

##### Detection and quantification limits

The method was developed to accommodate both negative and positive ESI polarity with the same mobile phase. Because the majority of the compounds ionised better in the negative ionisation mode the mobile phase was chosen to facilitate negative ionisation. This can be observed in the instrumental detection and quantification limits that are in the low ng L^−1^ (between 10 and 50 ng L^−1^) for most compounds analysed in negative mode while the compounds analysed in positive mode have IDL and IQLs in the hundreds of ng L^−1^ (Tables 1 and 2).

##### Linearity range

A set of 13 calibration solutions containing all analytes and internal standards were made up at the following concentrations: 0.01, 0.025, 0.05, 0.1, 0.25, 0.5, 1, 2.5, 5, 10, 25, 50 and 100 ng mL^−1^. These solutions were analysed and integrated using the QuantAnalysis software. For each analyte, the analyte/internal standard area ratio for the 13 calibration levels was compared and investigated by drawing up calibration curves. The *R*^2^ value for the resulting calibration curves is presented alongside their linear range in Table 1. Most analytes showed good linearity (interpreted from the *R*^2^) with varying linear ranges (from two to three orders of magnitude) highly dependent on individual analytes.

#### Targeted analysis of environmental samples with MAE/SPE-UHPLC-QTOF including full structural confirmation with commercially available reference standards

Environmental samples collected over 1-week-long sampling campaign were subjected to both quantitative and qualitative analysis. A summary for the quantified analyte levels in five different matrices is shown in Table 3**.** Several analytes were present at very high concentration levels in incoming and effluent wastewater. Other compounds showed an irregular occurrence pattern. For example, phenylbenzimidazolesulfonic acid (also known as Ensulizole or PBSA) was detected at high levels (> 1000 ng L^−1^) in influent and effluent wastewater with constant occurrence patterns. However, a spike in its concentration was observed on two consecutive days within the sampling week in the river water resulting in increased levels reaching 4000 ng L^−1^. This could be due to (accidental) disposal of larger quantities of PBSA upstream from the sampling point.

**Table 3:** Environmental data week average (results for 7 days sampling campaign with 24-h composite (wastewater) or grab (river water) samples)

Analyte	Wastewater influent (ng L^−1^)	Wastewater effluent (ng L^−1^)	River upstream (ng L^−1^)	River downstream (ng L^−1^)	Digested sludge (ng g^−1^)
2,4,5- & 2,4,6-trichlorophenol	2.5 ± 1.4	2.4 ± 1.0	0.1 ± 0.3	0.04 ± 0.16	57.5 ± 3.7
2-ethylhexanoic acid*	17,612.3 ± 21,685.4	417.0 ± 236.1	4290.5 ± 2277.5	3030.9 ± 1594.6	n.a.
2-naphthol	78.0 ± 20.5	9.3 ± 6.4	7.9 ± 9.2	6.7 ± 8.0	n.a.
4,4′-dihydroxybenzophenone	24.2 ± 4.2	15.2 ± 1.5	1.7 ± 0.4	2.0 ± 0.4	3.2 ± 0.19
4-benzylphenol	10.4 ± 6.0	18.2 ± 9.2	43.0 ± 64.9	28.3 ± 38.7	n.a.
4-chloro-3,5-dimethylphenol	8492.4 ± 3344.6	3975.2 ± 932.8	34.7 ± 13.7	100.8 ± 29.5	455.6 ± 7.5
4-chloro-3-methylphenol	2632.9 ± 903.7	43.3 ± 16.2	4.4 ± 1.5	3.3 ± 0.9	520.5 ± 16.6
4-n-nonylphenol	< MQL	31.2 ± 16.5	12.1 ± 7.9	13.3 ± 6.5	3.4 ± 0.06
4-n-octylphenol	n.d.	n.d.	n.d.	n.d.	n.d.
Atrazine	48.3 ± 4.1	45.3 ± 6.7	66.6 ± 5.2	59.5 ± 9.6	n.d.
Benzophenone-1	47.1 ± 14.6	6.8 ± 1.5	0.6 ± 0.3	0.8 ± 0.2	8 ± 0.6
Benzophenone-2	13.1 ± 2.5	10.6 ± 0.9	0.9 ± 0.6	0.9 ± 0.7	3.8 ± 0.4
Benzophenone-3	753.0 ± 129.2	44.7 ± 7.2	32.4 ± 34.1	4.5 ± 11.7	42.4 ± 5
Benzophenone-4	2711.5 ± 808.1	660.9 ± 157.7	41.6 ± 19.4	55.3 ± 27.9	33.1
Benzylparaben	n.d.	n.d.	n.d.	n.d.	0.44 ± 0.07
Bisphenol A	812.5 ± 52.2	23,215.9 ± 21,945.31	14.3 ± 8.5	12.9 ± 3.4	15,690 ± 542
BADGE-2-Cl	0.9 ± 0.67	0.31 ± 0.3	0.73 ± 0.38	0.77 ± 0.43	3.2 ± 0.07
Butylparaben	11.1 ± 4.9	1.3 ± 0.2	1.4 ± 0.7	1.7 ± 0.6	n.a.
Chlorothymol	n.d.	n.d.	n.d.	n.d.	5.6 ± 0.1
Dibutyl phthalate	49,208.3 ± 15,473.6	3909.9 ± 1069.8	943.0 ± 796.7	631.0 ± 365.0	n.a.
Ethylparaben;	143.3 ± 23.2	1.1 ± 0.3	2.6 ± 3.5	1.2 ± 1.3	5.34 ± 1.5
Galaxolide	164.5 ± 40.2	50.2 ± 18.7	11.0 ± 4.2	9.4 ± 2.7	n.a.
HBCD*	1.29 ± 0.84	0.73 ± 0.33	0.93 ± 0.88	0.57 ± 0.49	6.03 ± 1
MEHP	174.4 ± 319.4	47.0 ± 55.8	45.0 ± 24.4	38.8 ± 14.2	149 ± 3.4
Methylparaben	749.7 ± 136.8	2.8 ± 0.7	9.7 ± 9.1	5.8 ± 7.1	88.8 ± 2.7
Monobutyl phthalate	1305.4 ± 139.0	397.1 ± 52.5	18.0 ± 14.7	12.3 ± 9.7	49.5 ± 2.4
Musk ketone	n.d.	n.d.	n.d.	n.d.	n.a.
Padimate O	140.4 ± 42.0	94.6 ± 32.3	139.8 ± 147.8	248.5 ± 157.1	n.a.
Perfluorooctanesulfonic acid	4.5 ± 1.0	7.5 ± 2.8	11.9 ± 3.1	12.8 ± 3.7	n.d.
Perfluorooctanoic acid	10.0 ± 6.7	12.3 ± 7.6	12.6 ± 3.6	12.1 ± 4.5	4 ± 0.7
PBSA	2152.1 ± 484.1	1606.0 ± 473.9	3655. 4 ± 5504.6	298.2 ± 100.6	162 ± 27.7
Prochloraz	100 ± 62	5 ± 4.5	35.3 ± 32.8	22 ± 20.1	n.a.
Propylparaben	143.1 ± 23.3	4.7 ± 0.7	1.5 ± 1.2	1.4 ± 1.8	4.4 ± 0.5
Tetrabromobisphenol A	13.9 ± 11	1.67 ± 0.32	n.d.	n.d.	25.1 ± 1.4
Triclocarban	17.1 ± 5.1	8.5 ± 1.6	4.7 ± 3.4	3.4 ± 2.0	n.a.
Triclosan	589.0 ± 59.3	133.2 ± 14.6	19.8 ± 10.0	18.7 ± 6.2	n.a.
Vinclozolin	n.d.	n.d.	n.d.	n.d.	n.a.

Note: 1: based on triplicate extractions. 2: based on triplicate injections, for some analytes one injection had to be removed making *n* = 8 3: n.a. non analysed as compounds showed poor or no recovery from solid samples; *- results are only semi-quantitative dues to poor accuracy and precision

It was also interesting to note the low presence (< MQL) of the surfactant 4-n-nonylphenol in influent wastewater while its concentration in effluent wastewater was determined to be higher than > 30 ng L^−1^. That might be due to its formation during wastewater treatment as a result of decomposition of nonylphenol polyethoxylates [31]. Similar pattern was observed for BPA which concentrations were significantly higher in effluent than influent wastewater. It is suggested that the increase in concentration might be due to in situ degradation of conjugated metabolites (i.e. glucuronidates) as it has been previously observed for other compounds [32]. Nonetheless, in both cases, further investigation is required. Out of the 37 compounds investigated, only 3 compounds (n-octylphenol, musk ketone, vinclozolin) were not detected in studied environmental matrices, likely due to low sensitivity of the method towards these compounds.

Significant concentrations of 22 endocrine disruptors were also found when analysing solid samples (digested sludge). Concentrations ranged from 0.44 ng g^−1^ for benzylparaben to 15 μg g^−1^ for BPA. The observation of benzylparaben, along with chlorothymol, was of particular interest given that these compounds were not detected in wastewater and river water samples, highlighting the importance of investigating the presence of chemicals in solid matrices alongside water samples.

### Post-acquisition data mining for metabolite identification and quantification

The main advantage of LC-HRMS is the possibility of identifying compounds which were not included in the initial analysis (post-target or retrospective analysis) that can be achieved without the need for re-extraction/analysis (meaning reduction in the use of solvents, reagents and materials). This enables the investigation of newly identified compounds that are not yet integrated into the monitoring strategies currently in use such as compound’s metabolites (e.g. BPA sulphate, metabolite of BPA and 4-Cl-3-methylphenol sulphate, metabolite of 4-Cl-3-methylphenol).

As discussed in the Experimental section, the level system approach utilised in this study to identify and quantify metabolites with different levels of confidence was based on the work by Schymanski et al. [28]. Two confidence levels were investigated in this work:

- Level 1a: Metabolite structure confirmed by commercially available reference standards followed by full quantification (BPA sulphate, metabolite of BPA).
- Level 1b: Metabolite structure confirmed by reference compounds synthesised in vitro (4-Cl-3-methylphenol sulphate, metabolite of 4-Cl-3-methylphenol).

#### Example of a metabolite structure confirmed by commercially available reference standards followed by full quantification

**Fig. 4:**
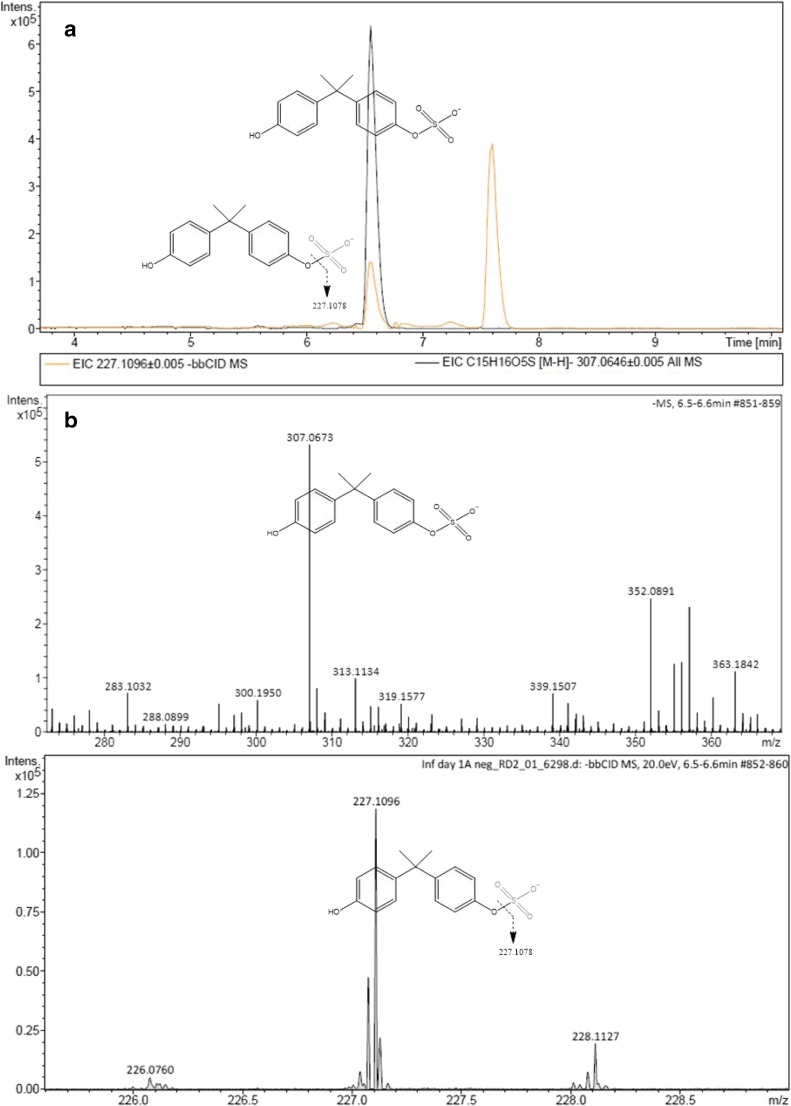
Detection and identification of BPA sulphated in wastewater by UHPLC-QTOF-MS. XICs at m/z 307.0646 and 227.1096 (0.005-Da mass-window width) (**A**). **B** (top) Low-energy (full-scan analysis) and (bottom) high-energy (bbCID mode) spectra and structures of the metabolite and fragment ion observed

Post-acquisition data mining of wastewater samples revealed a signal at m/z 307.0646 extracted from the total ion current corresponding to bisphenol A sulphate (elemental formula C_15_H_16_O_5_S with a mass error of 9 ppm) (Fig. 4) and a chromatographic peak at 6.8 min. Retention time of BPA sulphate was expectedly earlier than that of its parent compound BPA under the chromatographic conditions (reverse phase) due to the higher polarity of metabolite versus the parent compound. BPA was in fact eluted after 7.7 min. In order to confirm that the chromatographic peak corresponds with BPA sulphate, the MS identification workflow developed by Lopardo et al. [29] utilising retention time, mass accuracy, isotopic pattern and MS/MS fragments was employed (Fig. 4). A further level of confidence was added when results were compared to commercially available reference standard reaching the highest level of confidence as recommended by Schymanski et al. [28].

Subsequently, a calibration plot (*R*^2^ = 0.997) at eight concentration levels (each one replicated three times) ranging from the LOQ (1.39 ng mL^−1^) to 103.4 ng mL^−1^ was used to quantify BPA sulphate. LOD and LOQ were expressed as the concentration of BPA sulphate that give a signal to noise ratio of 3 and 10. Once HLB recoveries were assessed following the same protocol described in “Liquid matrix - solid-phase extraction” section method detection and quantification limits (MDL and MQL) were established as respectively 0.016 and 0.055 ng L^−1^. BPA sulphate was then found to be in wastewater at a concentration averaging at 2664 ng L^−1^ (Table 4).

**Table 4:** UHPLC-QTOF instrument and method performance parameter and BPA sulphate concentration in wastewater

Analyte	IS	Linearity Range [μg L^−1^]	*R* ^2^	Accuracy^1^ [%]	Precision^1^ [%]	IDL [μg L^−1^]	IQL [μg L^−1^]	Wastewater (influent) Method recovery [%]**	MDL [ng L^−1^]	MQL [ng L^−1^]	Average concentration in wastewater [ng L^−1^]***
Bisphenol A sulphate	4-chloro-3-methylphenol-d2	1.4–103.4	0.9972	98.3	2.1	0.4	1.4	63.7 ± 6.3	0.02	0.06	2663.9 ± 422

*Concentration levels: 0.1, 5 and 100 ng/mL used for precision and accuracy; **Based on duplicate extractions at two concentration levels; ***Based on wastewater samples collected over 7 days

#### Example of a metabolite structure confirmed by reference compound synthesised in vitro

**Fig. 5:**
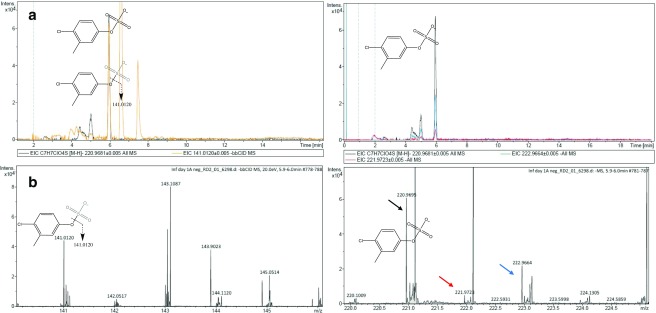
Detection and identification of 4-Cl-3-methylphenol sulphated in wastewater by UHPLC-QTOF-MS. XICs at m/z 220.9681 and 141.0113 (0.005-Da mass-window width) (**A**, left). XICs at m/z 220.9681, 221.9723 and 222.9664 (0.005-Da mass-window width) (**A**, right). **B** (left) High-energy (bbCID mode) and (right) low-energy (full-scan analysis) spectra and structures of the metabolite and fragment ion observed

Post-acquisition data mining of recorded mass spectra of wastewater samples revealed the presence of another metabolite that was previously discovered in in vitro HLM/S9 fraction experiments of 4-Cl-3-methylphenol simulating human liver metabolic processes [29]. A signal at m/z 220.9681 corresponding to 4-Cl-3-methylphenol sulphate (elemental formula C_7_H_7_ClO_5_S with a mass error of 6.4 ppm) was extracted from the total ion current of each wastewater chromatogram (Fig. 5) and a chromatographic peak at 5.9 min was found in all the samples analysed. Retention time of 4-Cl-3-methylphenol was expectedly earlier than the corresponding parent’s retention time under the chromatographic conditions (reverse phase, 7.5 min) due to the metabolite’s higher polarity than the parent compound. In order to confirm that the chromatographic peak corresponds with the 4-Cl-3-methylphenol sulphate, the workflow developed by Lopardo et al. [29] was employed as described above. Unfortunately, 4-Cl-3-methylphenol sulphate reference standard is not commercially available, which would allow for only tentative identification (level 2 in Schymanski et al. [28] of the metabolite. To solve this problem, and to provide full identification and further level of confidence, 4-Cl-3-methylphenol sulphate was synthesised in vitro by incubating 4-Cl-3methyl phenol with HLM/S9 fraction assay as described by Lopardo et al. [29]. The following criteria: retention time, mass accuracy and MS/MS fragmentation pattern, were utilised, as described above, to confirm the identity of 4-Cl-3-methylphenol sulphate. Although not fully quantitative, this approach allows for full identification of metabolites even in the absence of analytical standards.

## Conclusions

This study proposes an analytical framework for targeted analysis of selected EDCs in environmental matrices combined with the potential for retrospective identification and quantification of selected EDC metabolites. A new multi-residue LC-MS/MS method was developed for the analysis of diverse endocrine disruptors in environmental samples. IQLs observed were < 1 μg L^−1^ for almost all compounds with some of them showing IQLs below 0.1 μg L^−1^. MQLs achieved were < 1 ng L^−1^ for most of the compounds detected in aqueous matrices and < 1 ng g^−1^ for those detected in digested sludge. The results were similar to other studies employing different analytical techniques such as LC-TQD [15, 33–35]. However, the prospect of running, on the same dataset, retrospective analysis and/or untargeted screening in quest for new compounds of interest (i.e. new synthetic compounds, unknown metabolites, degradation products) makes the current method and others using high resolution mass spectrometry much more versatile [29, 36–38]. Analysis of environmental samples revealed the presence of 34 out of the 37 compounds investigated. In addition, several of them were found in digested sludge, which confirms that for a more comprehensive understanding of exposure patterns and environmental impact, solid analysis cannot be neglected. Furthermore, post-acquisition data mining of wastewater data allowed for identification and quantification of BPA sulphate and identification of 4-Cl-3-methylphenol sulphate confirming the great potential for retrospective analysis.

## Electronic supplementary material

ESM 1 (PDF 399 kb)
