# Supplementary material for: A new analytical framework for multi-residue analysis of chemically diverse endocrine disruptors in complex environmental matrices utilising ultra-performance liquid chromatography coupled with high-resolution tandem quadrupole time-of-flight mass spectrometry
Source: Anal Bioanal Chem. 2018 Nov 22;411(3):689–704. doi: 10.1007/s00216-018-1483-y (PMC6338708; doi:10.1007/s00216-018-1483-y)
Supplement: Supplementary file 1 — (PDF 399 kb) [file 216_2018_1483_MOESM1_ESM.pdf]

Analytical and Bioanalytical Chemistry

Electronic Supplementary Material

A new analytical framework for multi-residue analysis of chemically diverse endocrine disruptors in complex environmental matrices utilising ultra-performance liquid chromatography coupled with high resolution tandem quadrupole time-of-flight mass spectrometry

Luigi Lopardo, Axel Rydevik, Barbara Kasprzyk-Hordern

Table S1 Endocrine disruptors selected for this study, their structures and their physical-chemical properties

Group	Analyte	Formula	Water solubility [g/L] at pH 7 Temp 25 °C	Vapor pressure (Torr, at 25 °C)	pKa	LogP	CAS number
**Antimicrobial (Fungicide)**	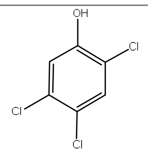 2,4,5-trichlorophenol	$C_6H_3Cl_3O$	0,15	0,0106	7.1	3.72	95-95-4
**Antimicrobial**	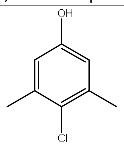 4-chloro-3,5-dimethylphenol	C_8H_9ClO	0,47	0,0177	9.76	3.35	88-04-0
**Antimicrobial**	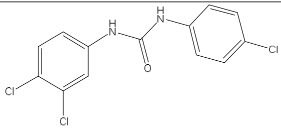 Triclocarban	$C_{13}H_9N_2OCl_3$	1,0E-4	6,67E-5	-0.02	6.07	101-20-2
**Antimicrobial**	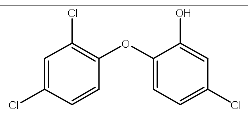 Triclosan	$C_{12}H_7Cl_3O_2$	1,3E-3	3,26E-5	7.8	5.34	3380-34-5
**Antiseptic**	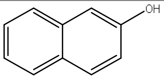 2-naphthol	$C_{10}H_8O$	0,49	1,62E-3	9.57	2.71	135-19-3
**Antiseptic**	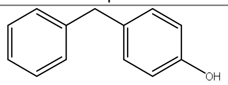 4-benzylphenol	$C_{13}H_{12}O$	0,10	1,54E-4	10.23	3.47	101-53-1
**Antiseptic**	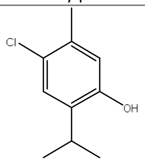 Chlorothymol	$C_{10}H_{13}ClO$	0,12	9,06E-3	10.16	4.22	89-68-9
**Flame Retardant**	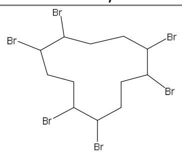 HBCD	$C_{12}H_{18}Br_6$	4,0E-5	7,80E-10	-	7.92	3194-55-6
**Flame Retardant**	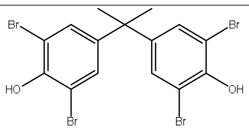 Tetrabromobisphenol A	$C_{15}H_{12}Br_4O_2$	3,8E-5	1,41E-7	8.5	9.69	79-94-7
**Musk**	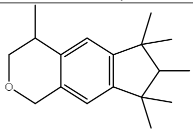 Galaxolide	$C_{18}H_{26}O$	0,024	4,14E-4	-	5.04	1222-05-5
**Musk**	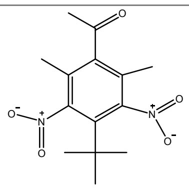 Musk ketone	$C_{14}H_{18}N_2O_5$	0,014	1,22E-5	-	2.51	81-14-1

**Paint Dryer**	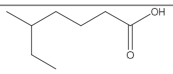<br>2-ethylhexanoic acid	$C_8H_{16}O_2$	343	0,0270	4.82	2.72	149-57-5
**Pesticide**	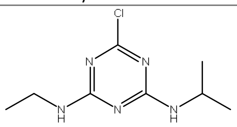<br>Atrazine	$C_8H_{14}ClN_5$	0,069	1,27E-5	2.27	2.64	1912-24-9
**Pesticide**	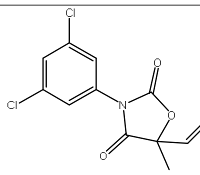<br>Vinclozolin	$C_{12}H_9Cl_2NO_3$	4,9E-4	1,15E-5	-3.43	3.27	50471-44-8
**Pesticide (Bactericide)**	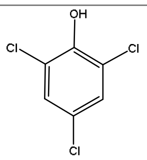<br>2,4,6-trichlorophenol	$C_6H_3Cl_3O$	0,32	0,0177	6.59	3.58	88-06-2
**Pesticide (Bactericide)**	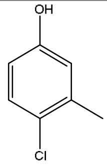<br>4-chloro-3-methylphenol	C_7H_7ClO	1,00	0,0335	9.63	2.89	59-50-7
**Pesticide (Fungicide)**	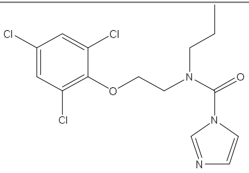<br>Prochloraz	$C_{15}H_{16}Cl_3N_3O_2$	0,02	4,02E-10	4.84	3.98	67747-09-5
**Plastic Additive**	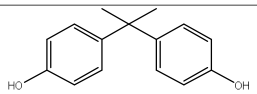<br>Bisphenol A	$C_{15}H_{16}O_2$	0.071	5,34E-7	10.29	3.64	80-05-7
**Plasticiser**	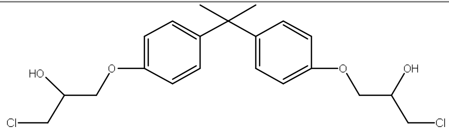<br>BADGE-2-Cl	$C_{21}H_{26}Cl_2O_4$	6,6E-3	1,56E-14	12.83	4.34	4809-35-2
**Plasticiser**	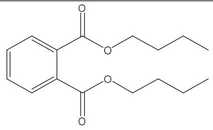<br>dibutyl phthalate	$C_{16}H_{22}O_4$	0,025	1,08E-4	-	4.8	84-74-2
**Plasticiser**	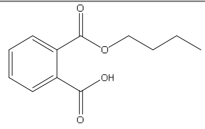<br>Monobutyl phthalate	$C_{12}H_{14}O_4$	478	6,40E-6	3.38	2.66	131-70-4
**Preservative**	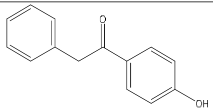<br>Benzylparaben	$C_{14}H_{12}O_3$	0,16	1,24E-6	8.18	3.57	94-18-8
**Preservative**	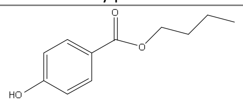<br>Butylparaben	$C_{11}H_{14}O_3$	0,54	3,56E-4	8.22	3.41	94-26-8
**Preservative**	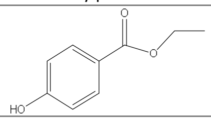	$C_9H_{10}O_3$	2,5	7,59E-4	8.31	2.39	120-47-8

	Ethylparaben						
**Preservative**	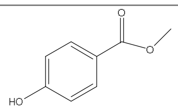 Methylparaben	$C_8H_8O_3$	5,6	5,55E-3	8.81	1.88	99-76-3
**Preservative**	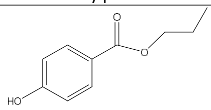 Propylparaben	$C_{10}H_{12}O_3$	1,2	9,30E-4	8.23	2.9	94-13-3
**Surfactant**	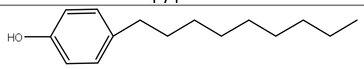 4-n-nonylphenol	$C_{15}H_{24}O$	0,02	8,53E-5	10.15	6.14	104-40-5
**Surfactant**	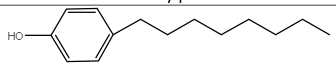 4-n-octylphenol	$C_{14}H_{22}O$	0,033	2,50E-4	10.15	5.63	1806-26-4
**Surfactant**	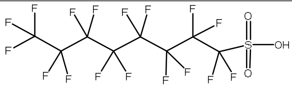 Perfluorooctanesulfonic acid	$C_8F_{17}O_3S$	7,5	-	-3.7	7.03	1763-23-1
**Surfactant**	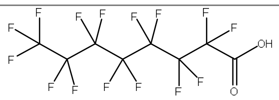 Perfluorooctanoic acid	$C_8HF_{15}O_2$	13	0,274	0.5	6.44	335-67-1
**UV-filter**	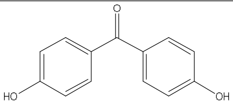 4,4'-Dihydroxybenzophenone	$C_{13}H_{10}O_3$	0,73	1,58E-8	7.67	2.63	611-99-4
**UV-filter**	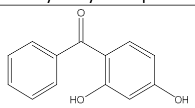 Benzophenone-1	$C_{13}H_{10}O_3$	0,51	2,84E-7	7.72	3.15	131-56-6
**UV-filter**	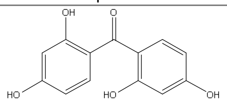 Benzophenone-2	$C_{13}H_{10}O_5$	3,2	6,69E-12	6.98	3.09	131-55-5
**UV-filter**	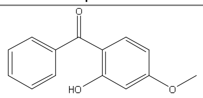 Benzophenone-3	$C_{14}H_{12}O_3$	0,13	5,26E-6	7.56	4	131-57-7
**UV-filter**	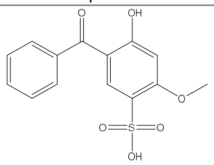 Benzophenone-4	$C_{14}H_{12}O_6S$	999	-	-0.7	0.99	4065-45-6
**UV-filter**	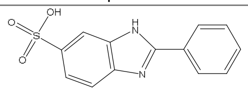 Phenylbenzimidazolesulfonic acid	$C_{13}H_{10}N_2O_3S$	1000	-	-0.20	1.5	27503-81-7
**UV-filter (Cosmetic)**	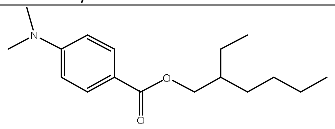 Padimate O	$C_{17}H_{27}NO_2$	4,7E-3	4,57E-6	2.39	6.15	21245-02-3

Table S2 Mass spectrometry parameters used in the identification of endocrine disruptors including mass error in ppm in all different matrices

Analyte	Formula	m/z		MRM transitions		Average mass error (ppm) (n=2)			
		Quantifier	Qualifier	Ion ratio	Collision energy (eV)	River water	Effluent WW	Influent WW	Digested sludge
2,4,5-trichlorophenol	$C_6H_3Cl_3O$	194.9177	158.9419	1.1	17	2.5	1.5	5	< 1
2,4,6-trichlorophenol	$C_6H_3Cl_3O$	194.9177	158.9419	1.1	17	2.5	1.5	5	< 1
2-ethylhexanoic acid	$C_8H_{16}O_2$	143.1078	127.1128	10000	18.6	< 1	< 1	2.6	2.6
2-naphthol	$C_{10}H_8O$	143.0502	115.0556	8	20	< 1	< 1	3.5	3.5
4,4'-Dihydroxybenzophenone	$C_{13}H_{10}O_3$	213.0557	93.0350	9	20	3.5	<1	<1	<1
4-benzylphenol	$C_{13}H_{12}O$	183.0815	106.0412	250	13	5	<1	5	<1
4-chloro-3,5-dimethylphenol	C_8H_9ClO	155.0269	119.0502	66.7	15	2	2	3.3	<1
4-chloro-3-methylphenol	C_7H_7ClO	141.0113	92.0267	1.28	14.5	<1	<1	2.8	<1
4-n-nonylphenol	$C_{15}H_{24}O$	219.1754	106.0430	14.9	17	4.5	3	1	2.5
4-n-octylphenol	$C_{14}H_{22}O$	205.1598	106.0430	3.8	17	3	1	3.5	1
Atrazine	$C_8H_{14}ClN_5$	216.1010	174.0561	0.9	17.4	4	2.5	4.5	9.5
Benzophenone-1	$C_{13}H_{10}O_3$	213.0557	135.0095	3.7	19	4	2	3.5	1.7
Benzophenone-2	$C_{13}H_{10}O_5$	245.0455	109.0301	3.5	12	2.4	2	3.6	1.2
Benzophenone-3	$C_{14}H_{12}O_3$	229.0859	151.0397	0.4	22	<1	2.6	5.6	5.6
Benzophenone-4	$C_{14}H_{12}O_6S$	307.0282	227.0721	2.5	27.4	3.3	3	2	<1
Benzylparaben	$C_{14}H_{12}O_3$	227.0714	136.0169	1	19	3.5	2.6	3.6	2
Bisphenol A	$C_{15}H_{16}O_2$	227.1078	211.0760	125	12	2.6	1	2	2.6
BADGE-2-Cl*	$C_{21}H_{26}Cl_2O_4$	411.1135	-	-	-	<1	1.5	<1	<1
Butylparaben	$C_{11}H_{14}O_3$	193.087	136.0169	2	17	3.5	2.5	3.5	2.5
Chlorothymol	$C_{10}H_{13}ClO$	183.0582	168.0348	166.7	14.5	4.4	2.7	5	<1
dibutyl phthalate	$C_{16}H_{22}O_4$	277.1445	208.0858	7.7	20	< 1	< 1	3.9	1.4
Ethylparaben	$C_9H_{10}O_3$	165.0557	136.0166	10	20	2.4	1.8	3	2.4
Galaxolide	$C_{18}H_{26}O$	259.2056	175.1126	0.5	17	< 1	1.5	3	5.4
HBCD*	$C_{12}H_{18}Br_6$	634.6436	-	-	-	2	< 1	2.3	-
MEHP	$C_{16}H_{22}O_4$	277.1445	233.1552	5.8	13.8	2.5	5	7.9	< 1
Methylparaben;	$C_8H_8O_3$	151.0401	136.0169	1.7	15	2	< 1	5	< 1
Monobutyl phthalate	$C_{12}H_{14}O_4$	221.0819	121.0300	3.3	25	2.7	2.7	2.7	< 1
Musk ketone	$C_{14}H_{18}N_2O_5$	293.1143	251.1039	6.3	26	2	2.4	5	7.9
Padimate O	$C_{17}H_{27}NO_2$	362.2115	166.0873	0.6	19	< 1	< 1	5.5	5.7
Perfluorooctanesulfonic acid	$C_8F_{17}O_3S$	498.9302	79.9580	200	31.5	3.8	5	< 1	< 1
Perfluorooctanoic acid	$C_8HF_{15}O_2$	412.9664	368.9783	0.1	11	4.3	4.3	1	< 1
Phenylbenzimidazolesulfonic acid	$C_{13}H_{10}N_2O_3S$	273.0339	193.0782	3.7	14	2.9	2.9	1.8	1.5
Prochloraz	$C_{15}H_{16}Cl_3N_3O_2$	376.0381	308.0035	0.04	12.8	< 1	1.6	3.8	5.7
Propylparaben	$C_{10}H_{12}O_3$	179.0714	136.0169	3.1	15	2.8	2.2	2.2	2.2
Tetrabromobisphenol A	$C_{15}H_{12}Br_4O_2$	538.7498	288.8870	3	13.8	< 1	2.2	1.7	1.5
Triclocarban	$C_{13}H_9N_2OCl_3$	312.9708	126.0115	16	20	< 1	2.9	< 1	< 1
Triclosan*	$C_{12}H_7Cl_3O_2$	286.9439	-	-	-	< 1	1.8	< 1	1
Vinclozolin	$C_{12}H_9Cl_2NO_3$	283.9887	140.0361	41.7	14	1.4	< 1	4.2	1.4

Note: 1 for starred compounds (due to poor fragmentation) only one MRM transition could be monitored.

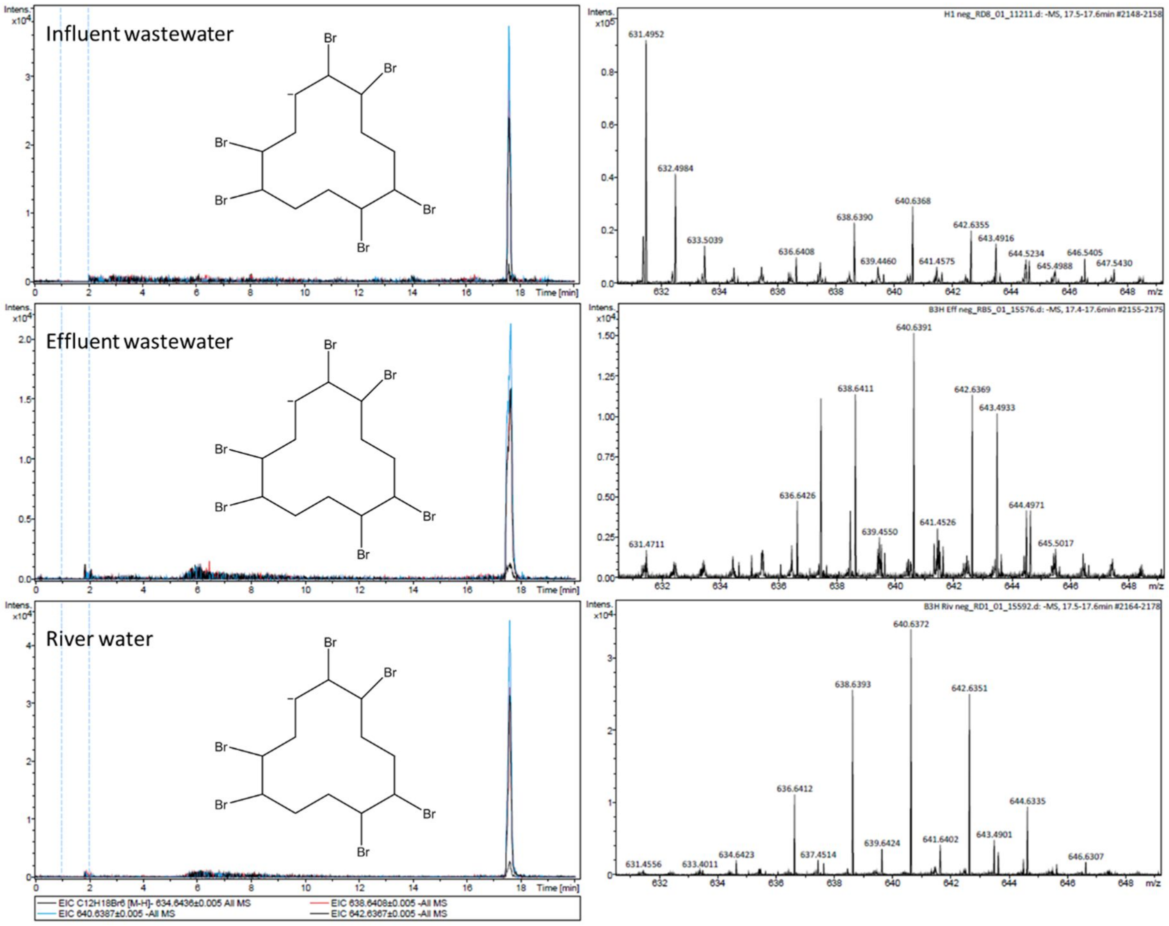

Fig. S1 Separation and identification HBCD in all analyzed matrices (River water, influent and effluent wastewater). XIC at m/z 634.6436 (0.005-Da mass-window width, black trace), at m/z 638.6408 (0.005-Da mass-window width, red trace), at m/z 640.6387 (0.005-Da mass-window width, blue trace) and at m/z 642.6367 (0.005-Da mass-window width, brown trace) in three different matrices (from top to bottom), and respective mass spectra of the peak eluted at 17.5 minutes
